# Design and Test of a Spatial Nanopositioner for Evaluating the Out-of-Focus-Plane Performance of Micro-Vision

**DOI:** 10.3390/mi14030513

**Published:** 2023-02-22

**Authors:** Ruizhou Wang, Heng Wu

**Affiliations:** 1 State Key Laboratory of Precision Electronic Manufacturing Technology and Equipment, Guangdong University of Technology, Guangzhou 510006, China; 2 Guangdong Provincial Key Laboratory of Cyber-Physical System, Guangdong University of Technology, Guangzhou 510006, China

**Keywords:** nanopositioner, micro-vision, out-of-focus-plane performance

## Abstract

Micro-vision possesses high in-focus-plane motion tracking accuracy. Unfortunately, out-of-focus-plane displacements cannot be avoided, decreasing the in-focus-plane tracking accuracy of micro-vision. In this paper, a spatial nanopositioner is proposed to evaluate the out-of-focus-plane performance of a micro-vision system. A piezoelectric-actuated spatial multi-degree-of-freedom (multi-DOF) nanopositioner is introduced. Three in-plane Revolute-Revolute-Revolute-Revolute (RRRR) compliant parallel branched chains produce in-focus-plane motions. Three out-of-plane RRRR chains generate out-of-focus-plane motions. A typical micro-vision motion tracking algorithm is presented. A general grayscale template matching (GTM) approach is combined with the region of interest (ROI) method. The in-focus-plane motion tracking accuracy of the micro-vision system is tested. Different out-of-focus-plane displacements are generated using the proposed nanopositioner. The accuracy degradation of the in-focus-plane motion tracking is evaluated. The experimental results verify the evaluation ability of the proposed nanopositioner.

## 1. Introduction

Micro-vision, consisting of a microscope and a camera, possesses the advantage of being a non-contact method with visualization capabilities [1,2,3,4,5,6,7]. The larger the eyepiece multiplier, the smaller the depth of field. Due to the small depth of field, micro-vision is generally employed to measure micrometer-scale or sub-micrometer-scale displacements in the focus plane. Several factors affect the in-focus-plane measurement accuracy, such as defocus blur, motion blur, and Gaussian blur. Unfortunately, out-of-focus-plane displacements are ubiquitously unavoidable. The relative distance between the lens of the microscope and the measured object always changes. The result causes different defocus blurs. Compared with macro-vision, the accuracy degradation of micro-vision is more prominent [6,7,8,9,10,11]. Out-of-focus-plane displacements of moving targets are more serious than those of stationary objects. Therefore, the defocus effect of micro-vision is worse for in-focus-plane motion tracking.

Spatial multi-degree-of-freedom (multi-DOF) nanopositioners play important roles in the fields of precision motion generation, measurement, machining, and manipulation [12,13,14,15,16,17,18]. Piezoelectric actuators (PEAs), compliant mechanisms (CMs), and parallel mechanisms (PMs) are widely selected elements to build nanopositioners [12,13,14,15,19,20]. PEAs generate sub-nanometer-scale displacements [12,13,14,15,18,19,20,21,22,23,24,25]. CMs transfer displacements or forces without any clearance or friction [12,13,14,15,19,20,22,24,26,27,28,29]. PMs enable the end-effector to have a higher motion generation precision and payload ability [12,13,14,15,19,20,26,27,28]. Combined with PEAs and compliant parallel mechanisms (CPMs), spatial nanopositioners can generate a motion with nanometer-scale accuracy. In-plane output displacements of the end-effector act as in-focus-plane tracking targets of micro-vision, and out-of-plane output displacements are used to evaluate the out-of-focus-plane performance of micro-vision.

This paper contributes a spatial nanopositioner and an evaluation approach for the motion tracking accuracy degradation characteristics of micro-vision. Firstly, the mechanical design approach of the spatial nanopositioner using six PEAs and a six-branched-chain CPM is proposed in Section 2. Secondly, the micro-vision system, utilizing the typical GTM and ROI methods, is presented to track in-focus-plane motion in Section 3. Thirdly, prototype tests measuring the in-focus-plane motion tracking accuracy degradation of the micro-vision system under different out-of-focus-plane displacements are conducted in Section 4. Finally, a brief conclusion is presented in Section 5.

## 2. Mechanical Design of the Spatial Nanopositioner

A spatial nanopositioner is proposed. A 6-Revolute-Revolute-Revolute-Revolute (6-RRRR) CPM acts as the mechanical unit of the nanopositioner. The 6-RRRR CPM consists of six parallel branched chains. Each branched chain is composed of four rotating pairs using notch flexure hinges. The first rotational pair, as the equivalent active pair, is denoted using R. The other three rotating pairs, as passive pairs, are represented using RRR. Therefore, every branched chain is labeled as RRRR. The 6-RRRR CPM possesses a two-in-one structural configuration of two layers. The upper layer is an in-plane 3-RRRR CPM. The lower layer is an out-of-plane 3-RRRR CPM. The two layers are connected using a metal plate. The end-effector of the nanopositioner connects the six RRRR branches directly. Six PEAs drive the six RRRR branches separately and act as the actuating unit of the nanopositioner.

### 2.1. In-Plane Motion Generation and Measurement

**Figure 1:**
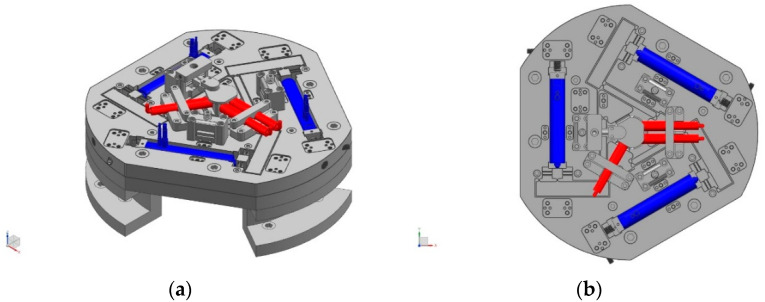
In-plane actuators and sensors of the proposed spatial nanopositioner. (**a**) 3D view; (**b**) top view.

The upper layer, namely, the in-plane 3-RRRR CPM, is composed of three RRRR branches. The three branches are located on the same plane. The in-plane three-degree-of-freedom (3-DOF) nanoscale-accuracy motion is generated. The end-effector acts as the tracking target of the micro-vision system. Three capacitive displacement sensors (CDSs) are used to measure the 3-DOF output displacements of the end-effector. Three PEAs (marked in blue) and three CDSs (marked in red) are shown in Figure 1.

### 2.2. Out-of-Plane Motion Generation and Measurement

**Figure 2:**
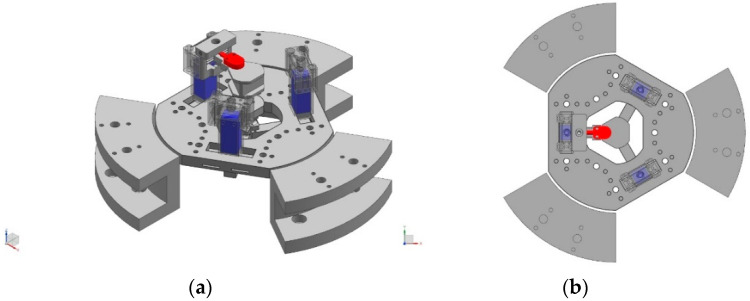
Out-of-plane actuators and sensors of the proposed spatial nanopositioner. (**a**) 3D view (**b**) top view.

The lower layer, namely, the out-of-plane 3-RRRR CPM, is composed of three RRRR branches. The three branches are located on three different planes. The plane of each out-of-plane RRRR branch is perpendicular to the same plane of the three in-plane RRRR branches. The out-of-plane motion is produced and added to the in-plane trajectory of the end-effector. One CDS is employed to measure the out-of-plane movement of the end-effector. Three PEAs (marked in blue) and one CDS (marked in red) are shown in Figure 2.

## 3. In-Focus-Plane Motion Tracking of Micro-Vision

In order to represent as many application cases as possible, typical calculation methods are selected for use in the micro-vision system. The focus plane of micro-vision is the calculation benchmark of out-of-focus-plane displacements. External measurement and image feature evaluation are two common methods to search the focus plane. Definition evaluation methods based on image features are mature, low-cost, and easy to implement.

### 3.1. Determination of the Focus Plane

The variance method is an algorithm used to characterize the difference in image sharpness values. The difference in the grayscale values of clear images is larger than that of fuzzy images. The pixel of the image is set as *M* × *N*. *F* is labeled as the result and expressed as follows:

(1) $$F=\sum_{i=1}^{M}\sum_{j=1}^{N}{\left[I(i,j)-\bar{\mu }\right]}^{2}$$

where *I*(*i*,*j*) denotes the grayscale value at point (*i*,*j*), and $\bar{\mu }$ represents the average grayscale value.

(2) $$\bar{\mu }=\frac{1}{MN}\sum_{i=1}^{M}\sum_{j=1}^{N}{I(i,j)}^{}$$

The variance evaluation function is unimodal and anti-noise. Based on the image sharpness function, the position of the clearest image is searched to determine the focus plane.

### 3.2. Grayscale Template Matching (GTM) Method

Typical template matching algorithms use the sum of squared differences (SSD) or normalized cross-correlation (NCC) to calculate the similarity. Let *S*(*x*,*y*) represent an image of size *M* × *N*, and *T*(*x*,*y*) denote a template image of size *m* × *n*. The similarity formula *D*(*i*,*j*) using the SSD algorithm is as follows:

(3) $$D(i,j)=\sum_{s=1}^{m}\sum_{t=1}^{n}{\left[S(i+s-1,j+t-1)-T(s,t)\right]}^{2}$$

where (*i*,*j*) represents the upper left corner. A subgraph with a size of *m* × *n* is taken to calculate the similarity to the template.

### 3.3. Region of Interest (ROI) Method

The typical ROI method is also used. Before the motion tracking, an original frame of the image is collected for template matching. The point (*u*_0_,*v*_0_) represents the central position of the original ROI area *m* × *n*. The original ROI area *R*_0_ of the original frame 0 is defined as follows:

(4) $$R_{0}=I(u_{0}-\frac{m}{2}:u_{0}+\frac{m}{2},v_{0}-\frac{n}{2}:v_{0}+\frac{n}{2})$$

The image of a new frame *i* is matched based on the ROI region of the previous frame *i* − 1. The point (*R_ui_*,*R_vi_*) of the new frame *i* represents the relative position in the ROI area of the previous frame *i* − 1. The point (*u_i_*,*v_i_*) of the new frame *i* represents the absolute position in the new frame *i* and is calculated as follows:

(5) $$\left(u_{i},v_{i})=(u_{i-1}-\frac{m}{2}+R_{ui},v_{i-1}-\frac{n}{2}+R_{vi}\right)$$

The updated ROI area *R_i_* of the new frame *i* is defined as follows:

(6) $$R_{i}=I(u_{i}-\frac{m}{2}:u_{i}+\frac{m}{2},v_{i}-\frac{n}{2}:v_{i}+\frac{n}{2})$$

The point (*u*,*v*) of every frame is acquired to calculate the in-focus-plane displacements.

## 4. Prototype Test of the Nanopositioner and Out-of-Focus-Plane Evaluation

Aluminum alloy 7075-T651 was selected as the material for the prototype of the presented spatial 6-RRRR CPM. Wire electrical discharge machining (WEDM) and computer numerical control (CNC) technology were used to fabricate the two 3-RRRR CPMs separately. The in-plane 3-RRRR CPM was equipped with three packaged PEAs (P-841.3B, Physik Instrumente GmbH, Karlsruhe, Germany). The packaged PEAs possessed an embedded strain gauge sensor (SGS), a closed-loop elongation of 45.0 μm, and a small size of Φ12 × 68 mm^3^. The out-of-plane 3-RRRR CPM was equipped with three naked PEAs (NAC2015-H28, Piezomechanik GmbH, Munich, Germany). The naked PEAs possessed a compact size of 10 × 10 × 28 mm^3^ and a long elongation of 42.3 μm. The four capacitance sensors consisted of three pillars and one flake (D-E20.200 and D-E30.200, Physik Instrumente GmbH). The nominal stroke of the four capacitive sensors was 200 μm, and the resolution was 6 nm. The positioning controller of the end-effector was built using a compact prototyping unit (MicroLabBox, dSPACE GmbH, Paderborn, Germany).

**Figure 3:**
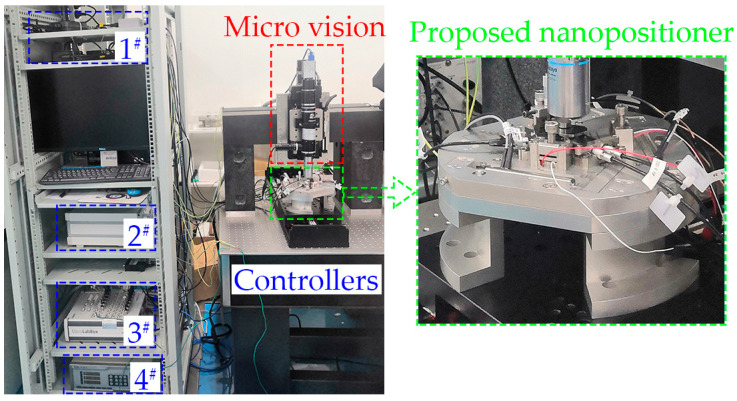
Prototype setup of the proposed nanopositioner, micro-vision system, and controllers.

The micro-vision system consisted of a microscope and a camera. The selected microscope had a magnification of 112.5 (Mitutoyo 50× objective, Navitar Inc., Rochester, NY, USA). The sensor of the selected camera had a resolution of 2448 × 2048 @ 75 fps, and a pixel pitch of 3.45 μm (Sony IMX250 CMOS, FLIR Systems Inc., Wilsonville, OR, USA). The microscope and camera were driven by a lifting sliding stage (KA050Z, Zolix Instruments Co., Ltd., Beijing, China). The resolution of this stage was 1 μm, and the positioning precision was better than ±3 μm. This stage was used to search for the focus plane of the micro-vision system. The equivalent pixel displacement relationship was calculated using a negative combined resolution and a distortion test target (R1L1S1N, Thorlabs Inc., Newton, NJ, USA). The calculated pixel displacement conversion relationship was 0.0311 μm/pixel. The whole experiment setup is shown in Figure 3.

As shown in Figure 3, four types of controllers were employed during the prototype test. The third controller (MicroLabBox) was the overall controller of the whole experimental system. The first controller connected the four capacitive sensors of the nanopositioner and the third controller. The second controller connected the six PEAs of the nanopositioner and the third controller. The fourth controller connected the lifting platform of the micro-vision system and the third controller.

### 4.1. Test Results of the in-Focus-Plane Motion Generation Ability

**Figure 4:**
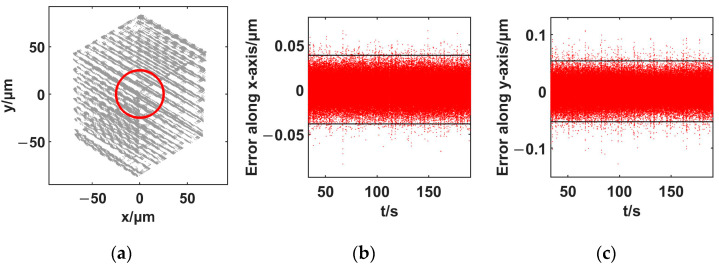
In-focus-plane motion generation ability using the proposed spatial nanopositioner. (**a**) planar workspace; (**b**) positioning error, *x* axis; (**c**) positioning error, *y* axis.

The in-plane workspace of the nanopositioner was tested. Then, an in-plane circular trajectory was generated using a PID controller. The results are shown in Figure 4.

As shown in Figure 4, the in-plane workspace of the nanopositioner is 140 × 170 μm^2^. For a circle with a diameter of 25 μm, the positioning error along the *x*-axis using the 3δ (δ: standard deviation) principle is 0.038 μm, and the 3δ error along the *y*-axis is 0.054 μm. The in-plane trajectory provides a standard in-focus-plane tracking target for the micro-vision system.

The area of the viewing field of the proposed micro-vision system is 76.1 × 63.7 μm^2^. The calculated pixel displacement conversion relationship is 0.0311 μm/pixel. The in-plane reachable workspace of the proposed nanopositioner is more than four times larger than the viewing field of the micro-vision system. The 3δ trajectory tracking precision of the nanopositioner is close to the identified displacement of one pixel of the micro-vision system.

### 4.2. Test Results of the Out-of-Focus-Plane Motion Generation Ability

**Figure 5:**
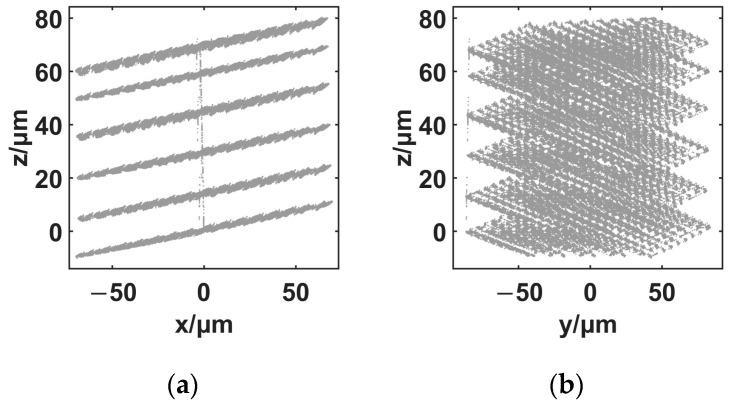
Out-of-focus-plane motion generation ability using the proposed spatial nanopositioner. (**a**) workspace, z-o-x plane; (**b**) workspace, z-o-y plane.

The out-of-plane stroke of the nanopositioner was tested. The results are shown in Figure 5.

As shown in Figure 5, the out-of-plane stroke of the nanopositioner is 90.4 μm. For every point in the area of the viewing field, 76.1 × 63.7 μm^2^, the corresponding out-of-plane stroke of the nanopositioner is more than ten times larger than the depth of focus of the micro-vision system. The out-of-plane motion of the nanopositioner is enough for the out-of-focus-plane excitation of the micro-vision system.

### 4.3. Test Results of the Out-of-Focus-Plane Performance

The field of view of the selected micro-vision system is 76.1 × 63.7 μm², and the sampling rate is 15 Hz. The in-focus-plane circular trajectory was generated, and different out-of-focus-plane harmonic displacements were added to the in-focus-plane trajectory. The motion tracking results of the circular diameter are shown in Table 1.

As shown in Table 1, out-of-focus-plane displacements changed the in-focus-plane measurement results of the micro-vision system. When the out-of-focus-plane displacement reached a threshold value, 7.737 ± 2.512 μm, the micro-vision system was unable to work.

### 4.4. Performance Comparison of Spatial Nanopositioners

The proposed spatial nanopositioner possesses a compact structure of Φ200 × 56 mm^3^, an in-plane workspace of 140 × 170 μm^2^, and an out-of-plane stroke of 90.4 μm. Compared with other nanopositioners [19,20,29], the presented nanopositioner has the ability to evaluate the out-of-focus-plane performance of micro-vision systems and can be easily embedded into these systems. The nanopositioner proposed in [20] can expand the actual application of optical alignment elements in projection lenses with 193 nm immersion lithography.

Additionally, the 3δ positioning accuracy of the proposed nanopositioner is satisfactory, being close to the identified displacement of one pixel of the micro-vision system. A comparison of the key performance indexes of the selected nanopositioners is shown in Table 2.

## 5. Conclusions

A spatial nanopositioner is proposed in this paper. The end-effector acts as the in-focus-plane measurement target of the micro-vision system. A 3-RRRR CPM is employed to generate in-plane motion. Another 3-RRRR CPM is used to generate different out-of-plane displacements to evaluate the out-of-focus-plane performance of the micro-vision system. The micro-vision system uses the typical GTM and ROI methods. The experimental results verify the accuracy degradation of the in-focus-plane motion tracking of the micro-vision system using different out-of-focus-plane displacements. The proposed nanopositioner possesses a motion generation ability for evaluating the out-of-focus-plane performance of micro-vision systems.

Future research will focus on the accuracy deterioration caused by high-frequency out-of-focus-plane displacements and the diffraction effect, and the real-time compensation or correction of the micro-vision system at the software level.

## Figures and Tables

**Table 1:** In-focus-plane tracking results excited by out-of-focus-plane displacements.

Out-of-Focus-Plane Displacements/μm	In-Focus-Plane Tracking Results/μm	In-Focus-Plane Accuracy Degradation/μm
−0.234 ± 2.518	28.474 ± 0.233	0.000
1.895 ± 2.515	28.606 ± 0.180	0.132
3.061 ± 2.515	28.638 ± 0.148	0.164
5.579 ± 2.513	28.892 ± 0.330	0.418
7.279 ± 2.513	29.027 ± 0.349	0.553
7.737 ± 2.512	cannot work	cannot work

**Table 2:** Comparison of key performance indexes of selected spatial nanopositioners.

Nanopositioner	Proposed	V. [19]	Z. [20]	C. [29]
Dimension/mm^3^	Φ200 × 56	250 × 250 × 80	Φ264 × 148	Φ150 × 143
In-plane workspace/μm^2^	140 × 170	40 × 40	80 × 80	8.2 × 10.5
Out-of-plane stroke/μm	90.4	80	60	13.2
*x*-axis, 3δ accuracy/μm	0.038	0.033	0.030	0.093 *
*y*-axis, 3δ accuracy/μm	0.054	0.033	0.030	0.081 *

Note: * = the maximum tracking error from the experimental result of bi-axial circular trajectories (3δ accuracy is not provided in this reference).

## Data Availability

Research data are available from the authors.
